# Aluminum Matrix Composites with Weak Particle Matrix Interfaces: Effective Elastic Properties Investigated Using Micromechanical Modeling

**DOI:** 10.3390/ma14206083

**Published:** 2021-10-14

**Authors:** Aharon Farkash, Brigit Mittelman, Shmuel Hayun, Elad Priel

**Affiliations:** 1 Department of Materials Engineering, Ben-Gurion University of the Negev, Bee’r-Sheva 84105, Israel; farkasha@post.bgu.ac.il (A.F.); hayuns@bgu.ac.il (S.H.); 2 Center for Thermo-Mechanics and Failure of Materials, Department of Mechanical Engineering, Shamoon Collage of Engineering, Bee’r-Sheva 84100, Israel; brigit@post.bgu.ac.il; 3 Nuclear Research Center Negev (NRCN), Materials Science Department, Bee’r-Sheva 84190, Israel

**Keywords:** AMCs, micromechanics, RVE, RAE

## Abstract

The impact of weak particle-matrix interfaces in aluminum matrix composites (AMCs) on effective elastic properties was studied using micromechanical finite-element analysis. Both simplified unit cell representations (i.e., representative area or volume elements) and “real” microstructure-based unit cells were considered. It is demonstrated that a 2D unit cell representation provides accurate effective properties only for strong particle-matrix bond conditions, and underpredicts the effective properties (compared to 3D unit cell computations) for weak interfaces. The computations based on real microstructure of an Al–TiB_2_ composite fabricated using spark plasma sintering (SPS) show that, for weak interfaces, the effective elastic properties under tension are different from those obtained under compression. Computations show that differences are the result of the local stress and strain fields, and contact mechanics between particles and the matrix. Preliminary measurements of the effective elastic properties using the ultrasonic pulse-echo technique and compression experiments support the trends observed in computational analysis.

## 1. Introduction

Aluminum matrix composites are attractive structural materials due to combining the unique characteristic of the aluminum alloy matrix with ceramic particles [1]. The aluminum matrix has a high strength-to-weight ratio compared with that of ferrous materials, and high ductility, good corrosion resistance, machinability, and processing flexibility, while ceramic particles are exceptionally strong [2]. By controlling the volume fraction of ceramic particles along with their size and shape, it is theoretically possible to tailor the effective thermal and mechanical properties to a specific engineering application. Effective properties govern the composite material response to external mechanical or thermal loading. This potential for a flexible and smart material design has led to over forty years of continuous research in both academia and industry [3].

Commonly used methods for AMC fabrication are casting (stir, squeeze, etc.), powder metallurgy, pressure infiltration, spray atomization, and co deposition [4,5]. When considering different fabrication methods, two main parameters are important to the composite’s effective properties. The first is the distribution of the reinforcing particles in the matrix, and the second is the resulting particle-matrix interface conditions [6]. While liquid-state fabrication processes may lead to the in situ formation of reinforcing particles in the melt, which can result in greater particle-matrix bonding strength, better control of particle distribution is obtained in solid-state processes in which the reinforcing particle and matrix particles are created ex situ, and then combined during the solid-state fabrication process. The different fabrication methods, advantages, and limitations can be found in a recent comprehensive review by Chak et al. [7].

To improve the strength of the aluminum matrix, the use of ceramic particles composed of SiC, Al_2_O_3_, or B_4_C is usually considered. TiB_2_ is also a promising candidate for aluminum reinforcement [8]. Nevertheless, while incorporating ceramic reinforcement to the aluminum matrix can result in increased yield and ultimate stress, it does not necessarily imply an increase in stiffness. The increase in AMC strength can be attributed to the localized stress fields among reinforcement particles, or the particles themselves acting as barriers for dislocation motion [9]. On the other hand, the increase in stiffness depends on the load transfer between particles and matrix [10]. In order to investigate microlevel local stress and strain fields, computational methods must be utilized. Such computations are commonly conducted on unit cell representations (representative area or volume elements—not to be confused with crystallographic unit cells) and are termed micromechanical computations. These models are able to describe mechanical fields resulting from particle-matrix interactions during mechanical or thermal loading. The unit cell approach was used to investigate the effective properties of porous materials [11] and various composites [12,13,14,15,16]. In [12], the unit cell approach was used to demonstrate that the reinforcing particle arrangement in the unit cell did not significantly influence the computed effective elastic properties of an Al–SiC composite. In [13], the method was used to show that the effective mechanical properties of an Al–B_4_C composite can be influenced by residual stress fields created during the fabrication by stir casting. Most computational studies reported in the literature assume that a perfect bond exists between particles and matrix [12,13,14,15,16,17]. Some studies modeled the bond strength by directly using cohesive-zone (CZ) approaches to investigate particle-matrix debonding, which initiates prior to the final failure of the material [15]. Nevertheless, it is commonly assumed that particle-matrix debonding only occurs after global effective yielding initiates [17]. The exact effect of very weak or nonexistent particle-matrix bonding strength on the effective elastic mechanical properties of AMCs is still not fully understood. This issue is especially important in AMCs, which are fabricated by a solid-state process that may result in materials with increased strength but decreased stiffness due to the weak particle-matrix bonding strength [18,19].

Most of the literature’s micromechanical computational models of AMCs employ a 2D representative area element (RAE) approach. The common assumption is that, for loading in the elastic range, a 2D approach is justified. It is not clear if this assumption is justified for AMCs that have weak particle–matrix interface strength. From a theoretical point of view, due to the particle-matrix interaction, 3D computations using a representative volume element (RVE) approach may be required. The main goal of the current study is to conduct a rigorous computational examination of the effective elastic properties of AMCs with weak particle-matrix interface strength. The computational study considers both simplified RVEs, which assume spherical particles, and “real” microstructures constructed directly from scanning electron microscopy (SEM) images of actual AMC specimens.

The highlights of the study are:

- A detailed comparison between effective elastic properties determined by RVE and RAE micromechanical models is conducted.
- Effective elastic properties for strong and weak particle-matrix interface conditions as a function of particle volume fraction are compared.
- The influence of loading mode (tension vs. compression) on the effective elastic properties for weak interface conditions is studied.

The manuscript is structured as follows. Section 2 outlines the micromechanical computational methodology utilized to study the effective elastic properties. RVE and RAE computational models for the representative microstructures, including RAE models constructed from SEM images, are described in Section 3. Computational results are presented in Section 4 and discussed in detail in Section 5. A summary and conclusions are given in Section 6.

## 2. Research Methodology

**Figure 1:**
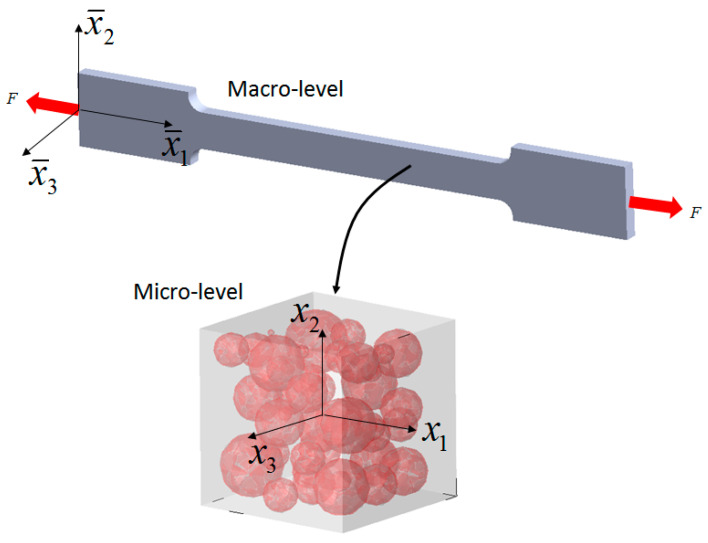
Uniaxial loading, macro- and microlevel representations.

The effective elastic properties of AMCs with strong or weak bonding are determined using both 2D and 3D micromechanical computations. The formulation of the boundary conditions that need to be applied in the micromechanical models is developed as follows:

Assume an AMC specimen that is statistically homogenous and isotropic is loaded in uniaxial tension or compression, as shown in Figure 1. Denoting the macrolevel stress and strain tensors as ${\bar{\sigma }}_{ij}\;\left(\bar{x}\right),\;{\bar{\varepsilon }}_{ij}\left(\bar{x}\right)$ strain and stress tensors at some material point $\bar{x}$ takes the following form:

(1) $${\bar{\sigma }}_{ij}=\left[\begin{array}{ccc} \sigma_{0} & 0 & 0\\ 0 & 0 & 0\\ 0 & 0 & 0 \end{array}\right]\;;\;{\bar{\varepsilon }}_{ij}=\left[\begin{array}{ccc} \varepsilon_{0} & 0 & 0\\ 0 & -\nu_{eff}\varepsilon_{0} & 0\\ 0 & 0 & -\nu_{eff}\varepsilon_{0} \end{array}\right]$$

where $\sigma_{0}\;,\;\varepsilon_{0}$ are the uniaxial stress and strain values resulting from the applied load, and $v_{eff}$ is the effective Poisson’s ratio of the composite. Standard homogenization theory requires that the averaged area or volume (either for a 2D or 3D representative cell) values of strain and stress at the microlevel must equal the pointwise macrolevel values [20].

(2) $${\bar{\sigma }}_{ij}\left(\bar{x}\right)=\frac{1}{V}\int_{V}\sigma_{ij}^{m}\left(x\right)dV\;{\bar{\varepsilon }}_{ij}\left(\bar{x}\right)=\frac{1}{V}\int_{V}\varepsilon_{ij}^{m}\left(x\right)dV$$

where x is the local coordinate vector at the microlevel (a unit cell with a volume of V), and $\sigma_{ij}^{m}\left(x\right)\;,\;\varepsilon_{ij}^{m}\left(x\right)$ represents the local stress and strain fields.

In order to maintain energetic consistency between the macro- and microscale, the microlevel stress and strain fields must comply with the Hill–Mandel condition [21]:

(3) $${\bar{\sigma }}_{ij}\left(\bar{x}\right):{\bar{\varepsilon }}_{ij}\left(\bar{x}\right)=\frac{1}{V}\int_{V}\sigma_{ij}^{m}\left(x\right):\varepsilon_{ij}^{m}\left(x\right)dV\Rightarrow \frac{1}{V}\int_{V}\sigma_{ij}^{m}\left(x\right):\varepsilon_{ij}^{m}\left(x\right)dV-{\bar{\sigma }}_{ij}\left(\bar{x}\right):{\bar{\varepsilon }}_{ij}\left(\bar{x}\right)=0$$

Using the Green–Gauss theorem, the area or volume integrals can be transformed into a boundary integral.

(4) $$\frac{1}{V}\int_{V}\sigma_{ij}^{m}\left(x\right):\varepsilon_{ij}^{m}\left(x\right)dV-{\bar{\sigma }}_{ij}\left(\vec{x}\right):{\bar{\varepsilon }}_{ij}\left(\vec{x}\right)=\frac{1}{V}\int_{S}\left(\sigma_{ij}^{m}n_{j}-{\bar{\sigma }}_{ij}n_{j}\right)\left(u_{i}-{\bar{\varepsilon }}_{ij}x_{j}\right)dA=0$$

Equation (4) results in displacement, traction, or mixed boundary conditions on the unit cell that automatically satisfies the Hill–Mandel condition [21].

As shown in Equation (4) the displacement boundary conditions on the unit cell depend on macro-level strain tensor $\;{\bar{\varepsilon }}_{ij}\left(\bar{x}\right)$ while the traction boundary conditions depend on macro-level stress tensor ${\bar{\sigma }}_{ij}\;\left(\bar{x}\right)$. As shown in Equation (1), the strain tensor components can be expressed as a function of applied axial strain $\;\varepsilon_{0}$ and the effective Poisson’s ratio $v_{eff}$, which is not known a priori. Under uniaxial loading, transverse stress components are zero. Since local stress and strain fields $\sigma_{ij}^{m}\left(x\right)\;,\;\varepsilon_{ij}^{m}\left(x\right)$ can be obtained from the unit cell, the following method determines the effective Poisson’s ratio $v_{eff}$ and the effective elastic modulus $E_{eff}$ of the composite:

1. Define some arbitrary uniaxial strain $\;\varepsilon_{0}$ within the elastic range, and assume an initial guess on effective Poisson ratio $v_{eff}$.
2. Define displacement boundary conditions on the unit cell by $u_{i}={\bar{\varepsilon }}_{ij}x_{i}$.
3. Solve the boundary value problem (BVP) on the unit cell and compute the lateral stress value.
4. If the absolute lateral stress value is smaller than some predefined small tolerance, the assumed value of $v_{eff}$ represents the composite; if not, Steps 1 to 4 need to be repeated.
5. Once the value of $v_{eff}$ is determined, the BVP solution can be used to compute $E_{eff}$ by $\sigma_{T}^{m}\;/\varepsilon_{0}$ with $\sigma_{T}^{m}$ being the average axial stress on the unit cell boundary.

A linear dependence exists between $v_{eff}$ and the transverse stress value. As a result, only two iterations of the above algorithm are required in order to identify $v_{eff}$ and compute $E_{eff}$.

The above algorithm was applied in the current study in order to determine the effective elastic properties for both RAE and RVE representations of AMC’s. The BVP was solved using the finite-element method utilizing commercial finite-element code ABAQUS/2021. The influence of particle-matrix interface conditions was directly taken into consideration in the micromechanical computation.

## 3. Micromechanical Computational Modeling

Two different computational microstructural unit cell representations were considered in this study.

1. A simplified unit cell composed of spherical ceramic particles of varying size randomly distributed in the aluminum cell matrix.
2. A unit cell constructed from SEM images of real Al–TiB_2_ fabricated using spark plasma sintering (SPS), previously investigated by the authors in [22].

**Figure 2:**
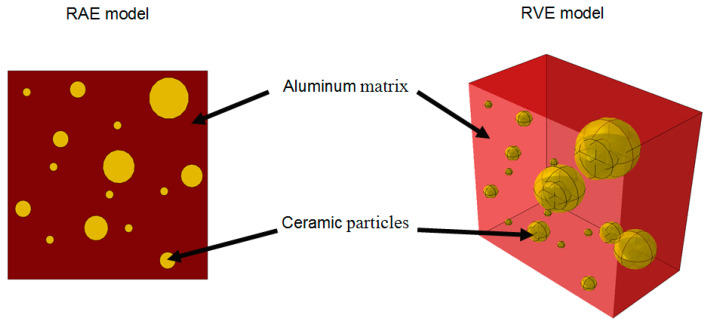
Microstructural model of RVE and RAE unit cells in which each RAE is a slice of a respective RVE.

In order to examine the difference between 2D and 3D representations of an AMC microstructure, the simplified geometries were considered. Each RAE was constructed as a discrete slice of the relevant RVE as demonstrated in Figure 2.

**Figure 3:**
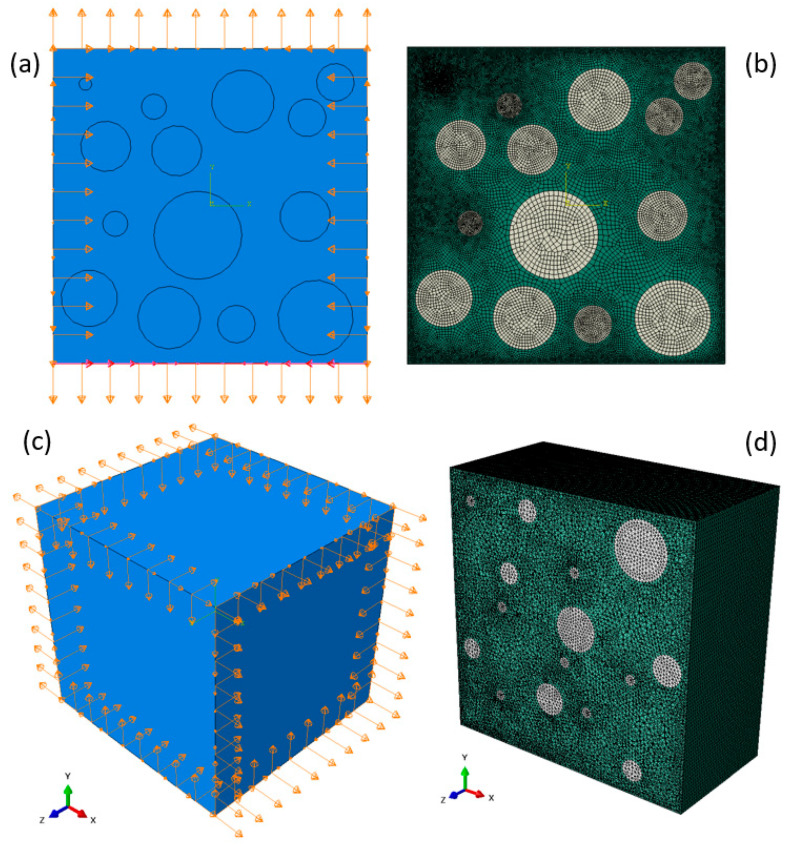
Representative boundary conditions and mesh for (**a**,**b**) 30% particle volume fraction under pure tension loading in a RAE unit cell and (**c**,**d**) 10% particle volume fraction under pure tension loading in a RVE unit cell. (**d**) Cut view in the middle of the RVE.

The RVE size for the simplified representation was 500 × 500 × 500 µm^3^ and contained particle volume fractions of 10%, 20%, and 30%. The RAE was meshed using triangular elements, while the RVE was meshed using tetrahedral elements; in both cases, full element integration was utilized. Two types of particle matrix interface conditions were considered: fully bonded and nonbonded. In nonbonded materials, there is no metallurgical or chemical bonding between the particles and the matrix, whereas in fully bonded materials, there is a strong metallurgical or chemical bonding between them. In order to numerically represent a strong bond, the nodes of each particle were tied to the nodes of the surrounding matrix, thus ensuring continuity in the displacement field. A weak interface was modeled using a nonpenetration condition in the normal direction with a coulomb-type friction in the tangential direction. Displacement boundary conditions were prescribed on all of the RVE/RAE boundaries according to (4) with a constant value of $\;\varepsilon_{0}=1e^{-4}$ used in all computations. To determine the effective properties using the previously described algorithm, computations using two values of $v_{eff}=0.2,0.5$ were conducted. An example of a representative mesh and boundary conditions for both RAE and RVE unit cells are shown in Figure 3.

The influence of the unit cell microstructure on the effective properties was further examined using RAE models that are based on SEM images of actual specimens of Al–TiB_2_. For this purpose, Al composites with 5%, 10%, and 15% TiB_2_ volume fractions were fabricated using SPS (commercially pure aluminum powder—E = 70 [GPa], v = 0.33 was used with TiB_2_ powder—E = 510 [GPa], v = 0.11). The average powder diameter was 32 and 7 µm for the Al and TiB2 powders, respectively. The details on the fabrication method of the Al–TiB_2_ composites are outlined in a recent publication by the authors [22]. The developed methodology for generating the computational model from the composite micrographs is presented here.

**Figure 4:**
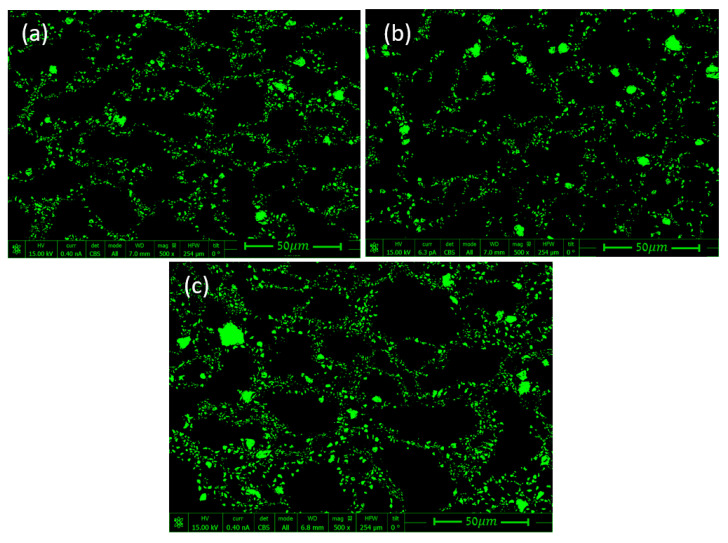
SEM micrographs of Al–TiB_2_ fabricated by SPS: (**a**) 5%, (**b**) 10%, and (**c**) 15% vol of TiB_2_ particles [22].

Composite specimens with 5%, 10%, and 15% volume fractions of TiB_2_ were polished, and their surface was then examined in SEM (see Figure 4). In the micrographs, Al and TiB_2_ phases are indicated in black and green, respectively. The SPS fabrication process in this case produced a “nonbonded” material, as the Al and TiB_2_ phases had no metallurgical or chemical bonding between them.

The micrographs in Figure 4 show that the TiB_2_ particles were heterogeneously arranged around large Al grains, unlike the previously presented RAE models (with arbitrarily distributed spherical particles). In order to convert the SEM images (Figure 4) into unit cell computational models, the following steps were taken:

1. Boundary detection of the TiB_2_ particles and segmentation of SEM images were conducted using a MATLAB [23] code written for this purpose.
2. The segmented .jpg image was converted into a .dxf file using the online Convertio [24] platform, thus transforming the image into a CAD sketch.
3. Digital scaling of the CAD sketch was performed using the SOLIDWORKS [25] program.
4. Scaled micrographs were imported into FE program ABAQUS [26].

When transforming the image into a CAD file, some of the features needed to be manually handled:

- Particles that were located along the edge were deleted (as there was a “nonbonded” interaction between particles and matrix, they may have been torn off the model under certain boundary conditions).
- Entities of very small scale from the SEM micrographs were also deleted.
- Incomplete contours of particles were manually completed.
- Particles of complicated shape that had been distorted during conversion were manually fixed or deleted.

**Figure 5:**
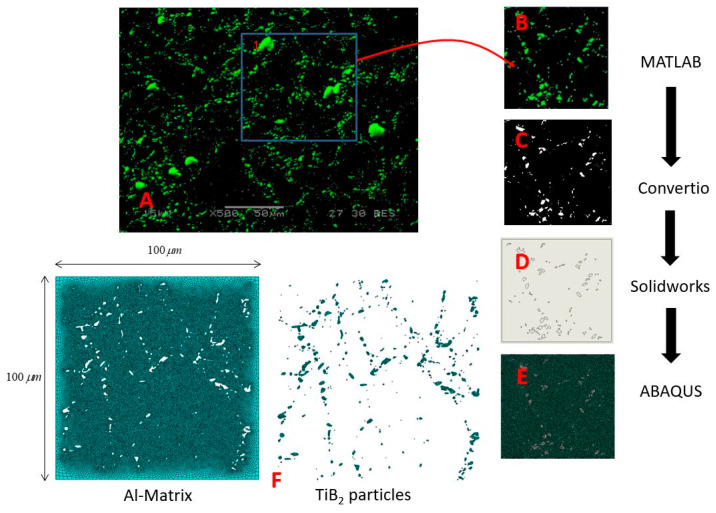
Process of converting the SEM image into a computational unit cell representation of the real AMC’s microstructure: (**A**) Back scatter (BS) images of the microstructure; (**B**) 100 × 100 µm^2^ section of BS image; (**C**) boundary detection and segmentation using MATLAB; (**D**) file conversion to DXF files and feature adjustment using CAD program; (**E**) export DXF file into ABAQUS and model creation, (**F**) computational unit cell showing both particle and matrix mesh.

These minor changes, in addition to digital deviations, create a small difference in the reinforcement volume fraction between the original micrograph and the RAE based on the image (see Appendix A). The whole process of generation of the RAE unit cell can be seen in Figure 5.

All computational unit cells were 100 × 100 µm^2^ in area. as it was determined that this is the smallest unit cell that could be taken without influencing the TiB_2_ volume fraction value (see Appendix B). For these unit cell dimensions, RAE models were constructed from different locations in the SEM image. By comparing the computed effective properties obtained at different locations (see Appendix A for an example), the particle distribution in the unit cell was verified not to influence the computed effective properties.

The boundary conditions, elastic properties of each constituent, interface conditions (for both bonded and nonbonded cases), element type, and integration scheme were similar to what is outlined above for the simplified unit cell representation. All computational unit cell models underwent standard verification procedures in which the number of degrees of freedom (elements) was increased until the relative error in both global values (such as strain energy and total force) and local values such as stress and strain was obtained.

## 4. Results

Two types of FE models were used to represent AMCs microstructure:

1. RVE models with randomly distributed spherical particles that were also randomly sized, and their respective RAE models that represented a discrete slice of the RVE.
2. RAE models constructed from real micrographs of Al–TiB_2_.

**Figure 6:**
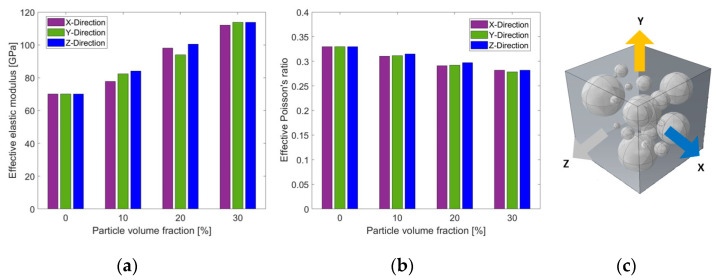
Effective elastic modulus (**a**) and Poisson’s ratio (**b**) obtained from RVEs under uniaxial tension (**c**) and fully bonded conditions for different particle volume fractions and loading directions.

First, the assumption of isotropy of the 3D representative models was examined. Effective elastic properties were obtained from RVE models at the three Cartesian directions (X, Y, Z) parallel to the RVE faces. The RVE was loaded in uniaxial tension in the X, Y, or Z directions. Each time, the effective elastic properties were calculated from the RVE for the direction parallel to the loading. Results are presented in Figure 6. Fully bonded interface conditions were defined between particles and matrix.

Figure 6 shows that the representative RVE models can be regarded as isotropic for all examined particle volume fractions. The maximal relative deviation that was obtained for both the elastic modulus and Poisson’s ratio was 8.1%. Figure 6 also shows clear trends of the relation between the effective elastic properties and the particle volume fraction. The elastic modulus increased with increasing particle volume fraction, while Poisson’s ratio decreased with increasing particle volume fraction.

**Figure 7:**
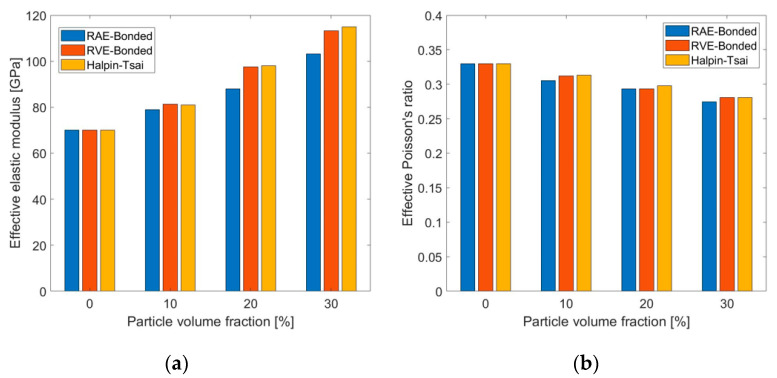
Comparison between 2D and 3D numerical results, and analytical results of effective elastic modulus (**a**) and Poisson’s ratio (**b**) for different particle volume fraction values.

Then, the 2D and 3D representative models were compared to examine the ability of RAE models to effectively predict the elastic properties. RAE models are easier to construct and require considerably fewer computational resources. The average values of the effective mechanical properties (with respect to the different directions) obtained for both RAE and RVE models are compared in Figure 7 for fully bonded interface conditions between particles and matrix. Since the RAE and RVE models were shown to be isotropic, and fully bonded conditions were assumed between matrix and particles, results were also compared to the analytical solution of the Halpin–Tsai model.

Figure 7 shows that there was good agreement between the results from the numerical models and the Halpin–Tsai estimations. The maximal relative deviation obtained between numerical and analytical results was about 9.7%. The maximal relative deviation between the RAE and RVE results was about 9%. Therefore, RAE models can predict the effective elastic properties for fully bonded interface conditions.

**Figure 8:**
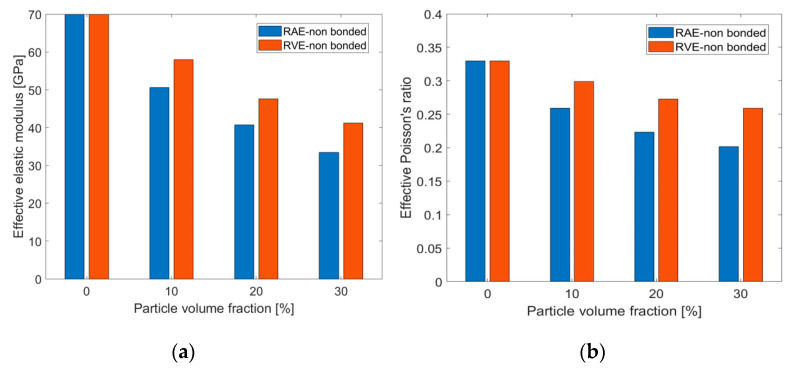
Comparison between effective elastic modulus (**a**) and Poisson’s ratio (**b**) obtained from 2D and 3D numerical models with nonbonded interface conditions for different particle volume fraction values.

In some AMC materials, such as the composite examined in this research, particles are not metallurgically bonded with the matrix, and transverse forces are transmitted by friction forces at the interface. There are no analytical solutions for these cases. RAE and RVE models with particle volume fractions of up to 30% were used to examine the influence of friction coefficient µ on the effective elastic properties. Friction coefficients of µ = 0–0.4 were considered. Computations showed that µ had negligible influence on the resulting effective elastic properties for particle volume fractions within the examined range due to the small relative sliding between particles and matrix under the elastic loading range. Therefore, only interface conditions of µ = 0 were used to examine their influence on the effective elastic properties for the nonbonded case. The average properties are presented in Figure 8. As before, the models were loaded in uniaxial tension. Isotropy was examined in the nonbonded models by calculating the effective properties for uniaxial tension in the X, Y, and Z directions. As in the fully bonded case, similar results were obtained in all three directions, so that isotropy could be assumed.

Figure 8 shows that the relation between the elastic modulus and the particle volume fraction is the opposite of the one obtained in the fully bonded case: the effective elastic modulus decreased as the particle volume fraction increased. This trend may be attributed to the fact that, when the interface was not bonded, there was no force transfer between particles and matrix. Poisson’s ratio shows a similar trend to the elastic modulus in Figure 8. In addition, there was considerable difference between the RAE and RVE results.

The presented results so far are based on RAE and RVE models for a simplified representative microstructure. These results show that the RAE unit cell representation underestimates the effective properties for composites with weak particle-matrix bonds. Nevertheless, the overall trends for this type of AMCs as a function of particle volume fraction can still be investigated by utilizing an RAE unit cell.

To better understand the interplay between particles and matrix at the microlevel, a more realistic microstructural representation is required. To this end, RAE models that were constructed from the SEM images of Al–TiB_2_ specimens were investigated. The influence of the conditions at the interface between particles and matrix (fully bonded vs. nonbonded) and the loading (tension vs. compression) on the effective elastic properties was examined.

To compare the different cases, we define the relative effective modulus and Poisson’s ratio as follows.

(5) $$E_{eff}^{R}=\frac{E_{eff}\left(A_{{}_{f}}^{p}[\%]\right)}{E_{eff}\left(A_{{}_{f}}^{p}=0\right)}\;;\;v_{eff}^{R}=\;\frac{v_{eff}\left(A_{{}_{f}}^{p}[\%]\right)}{v_{eff}\left(A_{{}_{f}}^{p}=0\right)}$$

**Figure 9:**
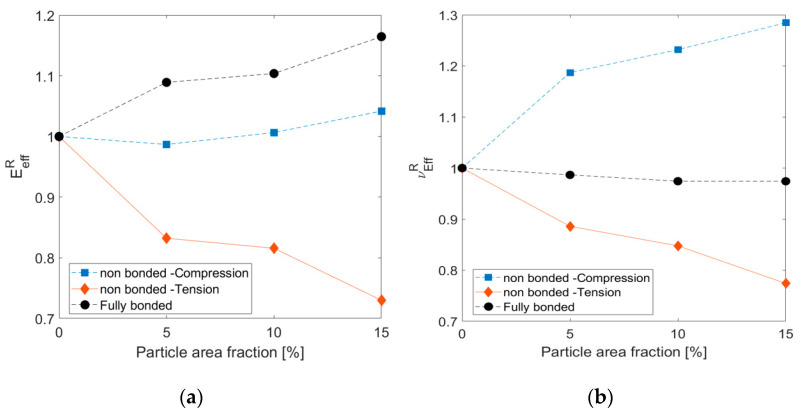
Relative effective elastic modulus (**a**) and Poisson’s ratio (**b**) for nonbonded interface for different particle volume fraction values under different loading and interface conditions compared to fully bonded case.

The fully bonded case under tension is compared with the nonbonded case under both tension and compression in Figure 9. In the fully bonded case, there is no difference between the effective properties obtained in tension and compression.

Figure 9 shows that the calculated properties from the fully bonded models showed similar trends to the results obtained by simplified representations and the theoretical Halpin–Tsai model. The relative value of the effective elastic modulus increased with the particle area fraction, as expected from the rule of mixtures (so the composite material properties lay between those of the matrix and the particles). On the other hand, the effective properties calculated from the nonbonded cases showed a different trend. Under tension loading, both the elastic modulus and Poisson’s ratio decreased, obtained in the simplified RVE computations. However, compression loading increases Poisson’s ratio with the particle volume fraction, and the elastic modulus trend is unclear.

### Partial Validation of Computational Findings

Computational analysis revealed that, for composites with weak particle-matrix interface strength, the effective elastic properties are both a function of the particle volume fraction and largely influenced by loading mode (i.e., tension or compression). To validate the computational findings, preliminary measurements of the effective elastic properties of Al–TiB_2_ were performed. Two types of measurements were conducted:

1. Ultrasonic pulse-echo technique.
2. Compression testing.

**Figure 10:**
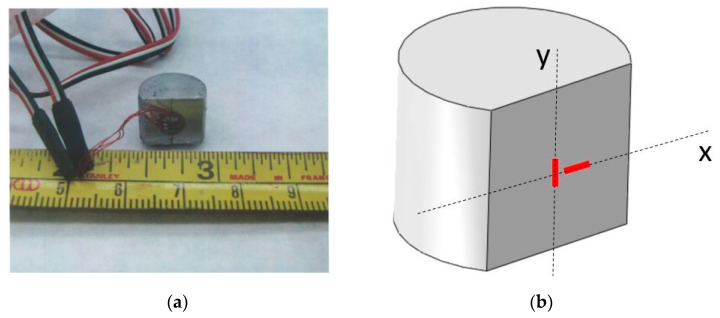
Compression specimen of Al–TiB_2_ composite fitted with axial and transverse strain gages (**a**) strain gage directions with respect to loading direction (**b**) (y loading direction, x transverse direction).

The ultrasonic pulse-echo technique is an accepted method for the evaluation of the elastic properties of different materials and composites [27,28]. Compression testing is commonly used to determine the elastoplastic behavior of metallic materials and composites [22]. Determining the elastic properties in compression requires specimens with large aspect ratios (as suggested in the ASTM E9-09 standard). The specimen described in [22,29,30] and utilized in this study is not suited for standard measurements of effective elastic properties. To overcome this difficulty, each cylindrical specimen was cut on one of its sides and fitted with two strain gages (type 060WT by Micro-Measurements Ltd., Wendell, NC, USA) in the axial and transverse directions, as shown in Figure 10.

**Figure 11:**
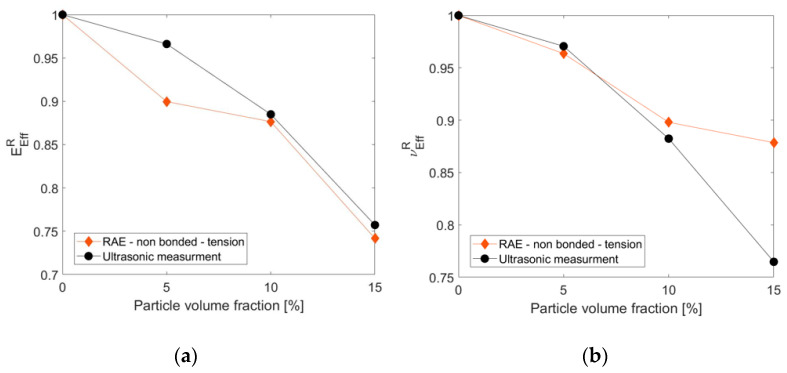
Comparison between measurements (pulse-echo technique) and predicted relative effective elastic modulus (**a**) and Poisson’s ratio (**b**) (RAE computations under tension loading) for different particle volume fractions.

The axial strain measurement was used to compute the compressive effective modulus of the composite, while the ratio between axial and transverse strain measurements was used to compute the effective Poisson’s ratio. Each measurement was repeated three times to ensure the repeatability of the results (standard deviation was in the ranges of 1.2–6.3 [GPa] and 0.0005–0.0062 for the effective elastic modulus and Poisson’s ratio, respectively). Comparing the measurements to the computed relative effective properties showed that the ultrasonic testing measurements agreed with the predicted effective properties trend under tensile loading, as seen in Figure 11.

**Figure 12:**
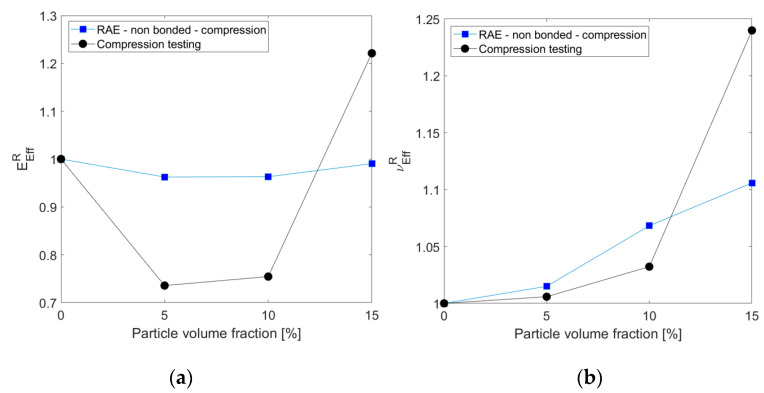
Comparison between measurements (compression testing) and predicted (RAE computations under compression loading) relative effective elastic modulus (**a**) and Poisson’s ratio (**b**) for different particle volume fractions.

Compression measurements are in general agreement with the predicted effective properties trend under compression loading, as seen in Figure 12.

## 5. Discussion

**Figure 13:**
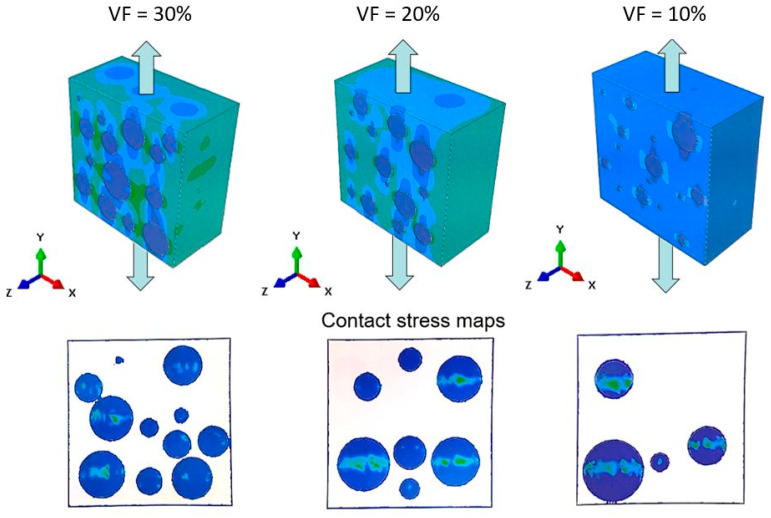
Contact-stress maps of RVE with nonbonded interface conditions for various particle volume fractions.

As discussed in the introduction, most studies that investigate the mechanical response of AMCs at the microscale employed 2D unit cell representations. The obtained results in this study demonstrate that this assumption is valid, even in the elastic loading range, only if a strong bond exists between particles and matrix. For weak bonds, 2D representation results in an underprediction of the effective elastic modulus. This result may be attributed to the difference in contact areas between RAE and RVE models, which influences the areas of interaction between particles and matrix. For example, contact stresses between particles and matrix for the RVE case are shown in Figure 13.

Figure 13 shows that, under tensile loading, contact stresses are generated in a circumferential pattern around each particle. For the RAE representation, the contact area is limited to a small section along the diameter of the particles transverse to the loading direction. These differences in contact-stress values can explain the differences in the computed effective elastic properties.

**Figure 14:**
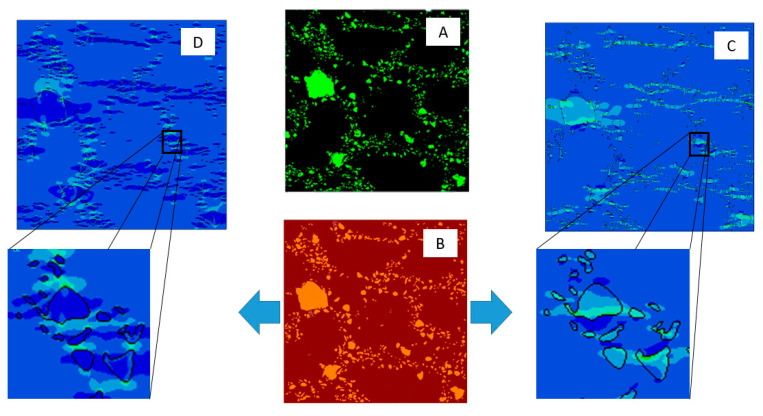
Local stress transfer across particles under tensile loading for fully bonded and nonbonded interaction: (**A**) original SEM image, (**B**) computational RAE, (**C**) fully bonded stress field in tensile direction, (**D**) nonbonded stress field in tensile direction.

Computations demonstrated that the effective elastic modulus decreases with the addition of reinforcing particles. This can be explained by observing stress fields near the particle-matrix interface for the bonded and nonbonded cases, as shown in Figure 14.

Figure 14 C,D show that high stress values developed in TiB_2_ particles for the bonded case. In the nonbonded case, the stress field inside the particles was small except at local points that were transverse to the loading direction. These lateral stresses are seen at points where the matrix pushed against the particle due to the transverse strain that developed as a result of the elastic deformation in the loading direction. Although the TiB_2_ particles were not bonded to the matrix, they should not be viewed as voids. As shown in Figure 14D, particles inhibited the lateral elastic deformation of the matrix, thereby influencing the effective Poisson’s ratio and thus the effective elastic modulus.

**Figure 15:**
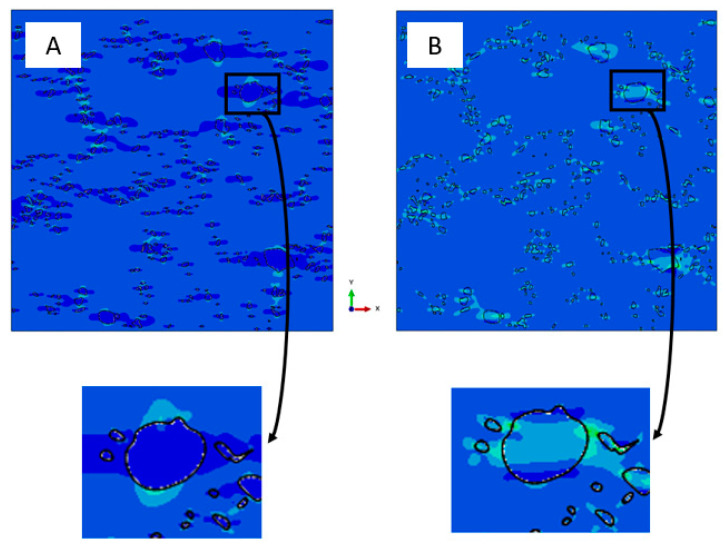
Local stress field in the X direction under (**A**) tension and (**B**) compression for nonbonded case.

Computational results also demonstrated that, for the nonbonded case, effective elastic properties also depend on the loading direction. To understand the reason for this dependency, we can again examine the local stress fields for the “nonbonded” case under tensile and compression loading, as shown in Figure 15.

Figure 14D and Figure 15A show that, while under tension, particles influence the effective properties only by hindering the lateral deformation and by the small contribution of the frictional forces at the contact areas. Under compression loading, particles can transmit the compressive force between adjacent areas of the matrix. This force is not fully transmitted due to the ability of the matrix to deform below and above the particle, with little resistance (except from frictional forces at the interface). This explains why the effective modulus under tension decreases with the increase in particle volume fraction, whereas it is almost not affected by the particle volume fraction under compression. As demonstrated in this study, RAE models introduce some error in the determination of the effective elastic properties for the nonbonded case, as shown in Figure 8. As a consequence, the comparison to the experiments was only used to validate the discovered trends in this study.

Although the current study deals with effective elastic properties, RAE computations can also begin to explain results reported by the authors of this manuscript in [22] which examines the AMC’s plastic range. Even for the nonbonded case, the yield stress of the Al–TiB_2_ composite increased with increasing values of the TiB_2_ volume fraction (up to 10%). This can be attributed to TiB_2_ particles acting as barriers for dislocation movement, but could also be related to the local stress and strain fields that develop due to the particle-matrix interface. As shown in Figure 14D and Figure 15, even for the nonbonded case, large stress gradients developed in the Al matrix near the TiB_2_ particle or between groups of TiB_2_ particles. These large stress gradients can also hinder dislocation motion and effectively increase the composite yield stress.

## 6. Summary and Conclusions

Micromechanical modeling was used to investigate the effective elastic properties of AMCs that can be statistically considered to be homogeneous and isotropic. Both strong and weak particle-matrix interface conditions were considered using both a simplified unit cell and real microstructural representations. Emphasis was given to cases where the interface was weak since no general analytical solution exists for estimating the effective elastic modulus and Poisson’s ratio.

Numerical results demonstrated that 2D computations provide an accurate estimation of effective elastic properties (compared to 3D representations) only if a strong particle-matrix interface can be assumed. For cases in which the interface is weakly bonded or nonbonded, the 2D micromechanical computations result in an underestimation of the effective properties, which should be taken into consideration.

For real microstructures, effective elastic properties depend on both the particle volume fraction and the loading direction. Under tension, the effective elastic modulus and Poisson’s ratio decreased for an increasing particle volume fraction. Under compression, the effective elastic modulus did not seem to change with increasing particle volume fraction, while the effective Poisson’s ratio seemed to increase. The local mechanical interaction between particles and matrix existed even for nonbonded conditions. As a result, the known analytical relations for the effective modulus of voided materials are not appropriate for predicting the effective properties.

These findings were examined by comparing the predicted variations in effective properties under the tension and compression of Al–TiB_2_ composites. Effective properties were measured using both the pulse-echo technique and mechanical compression testing. Initial experiments demonstrated the general trends observed in computational analysis. It is yet not clear if ultrasonic testing can be regarded as representative of tensile loading conditions or that computational findings should be compared to standard tensile tests.

## Figures and Tables

**Figure A1:**
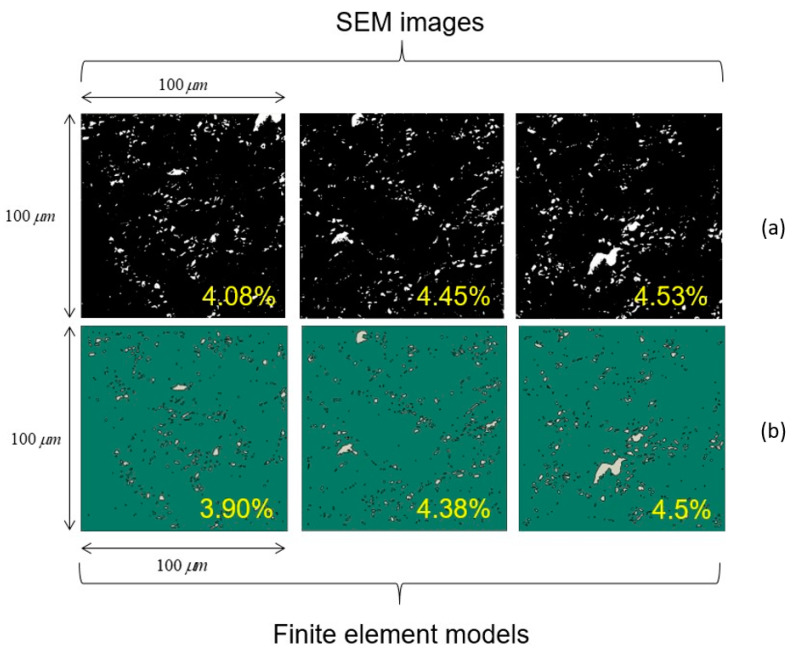
Comparison between particle area fractions computed for the (**a**) original SEM image and (**b**) unit cell representation.

**Figure A2:**
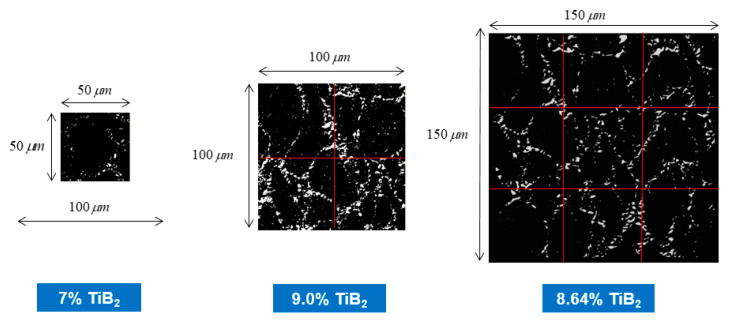
Example of particle area fraction obtained for increasing unit cell size for Al–TiB2 composite.

**Figure A3:**
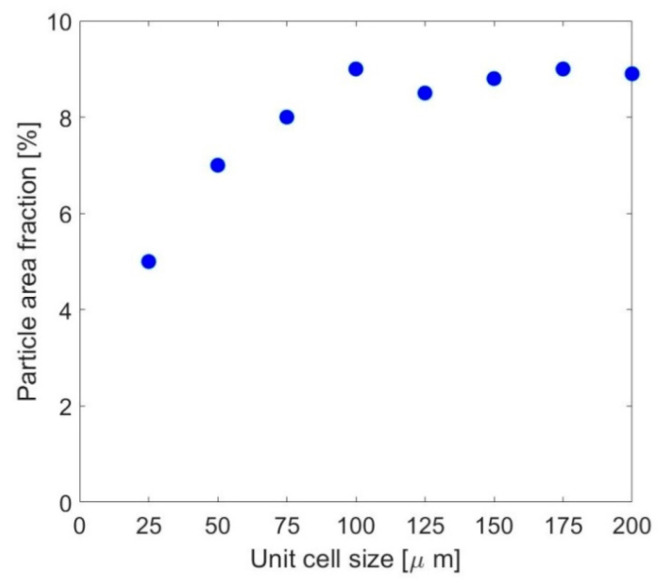
Particle area fraction as a function of unit cell size.

## Data Availability

Data is contained within the article.

## Appendix A. Estimation of Errors in Microstructural Representation

The computational methodology utilized in this study necessitates using SEM images to construct the microstructure geometry. The construction is based on image analysis and may introduce errors, especially with regard to the particle area fraction, which can influence the predicted effective elastic properties. To check the accuracy of the unit cell construction, the particle area fraction from the original SEM image was compared to the resulting computational unit cell image. This process was conducted for several different SEM images at different locations in the specimen. Figure A1 shows an example of such a comparison.

Analysis showed that the maximal difference in particle area fraction between the computational unit cell and the original SEM image is about 0.4%. This error does not significantly influence the predicted effective elastic properties.

## Appendix B. Limitations on Computational Unit Cell Size

The size of the unit cell that should be used in computational analysis is inherently linked to the relevant material microstructure. For the computed effective properties to accurately represent the bulk composite response, the particle area fraction derived from SEM images should remain constant for increasing unit cell dimensions. To verify this, on the basis of a 300 × 250 µm^2^ SEM image, several unit cells with different cell sizes were generated as shown in Figure A2.

The value of particle area fraction as a function of unit cell size is shown in Figure A3:

Figure A3 demonstrates, for unit cell sizes smaller than 100 × 100 µm^2^, the particle area fraction; as a result, effective elastic properties are unit-cell-size-dependent. Therefore, in the current study, all unit cells were equal to or larger than 100 × 100 µm^2^.
